# MicroRNA expression after ionizing radiation in human endothelial cells

**DOI:** 10.1186/1748-717X-5-25

**Published:** 2010-03-26

**Authors:** Mechthild Wagner-Ecker, Christian Schwager, Ute Wirkner, Amir Abdollahi, Peter E Huber

**Affiliations:** 1 Department of Radiation Oncology, German Cancer Research Center and University of Heidelberg Medical Center, Heidelberg, Germany; 2 Center of Cancer Systems Biology, NASA Specialized Center of Research, Caritas St, Elizabeth's Medical Center, Tufts University School of Medicine, Boston, MA 02135, USA

## Abstract

**Background:**

Endothelial cells (EC) in tumor and normal tissue constitute critical radiotherapy targets. MicroRNAs have emerged as master switchers of the cellular transcriptome. Here, we seek to investigate the role of miRNAs in primary human dermal microvascular endothelial cells (HDMEC) after ionizing radiation.

**Methods:**

The microRNA status in HDMEC after 2 Gy radiation treatment was measured using oligo-microarrays covering 361 miRNAs. To functionally analyze the role of radiation-induced differentially regulated miRNAs, cells were transfected with miRNA precursor or inhibitor constructs. Clonogenic survival and proliferation assays were performed.

**Results:**

Radiation up-regulated miRNA expression levels included let-7g, miR-16, miR-20a, miR-21 and miR-29c, while miR-18a, miR-125a, miR-127, miR-148b, miR-189 and miR-503 were down-regulated. We found that overexpression or inhibition of let-7g, miR-189, and miR-20a markedly influenced clonogenic survival and cell proliferation per se. Notably, the radiosensitivity of HDMEC was significantly influenced by differential expression of miR-125a, -127, -189, and let-7g. While miR-125a and miR-189 had a radioprotective effect, miR-127 and let-7g enhanced radiosensitivity in human endothelial cells.

**Conclusion:**

Our data show that ionizing radiation changes microRNA levels in human endothelial cells and, moreover, exerts biological effects on cell growth and clonogenicity as validated in functional assays. The data also suggest that the miRNAs which are differentially expressed after radiation modulate the intrinsic radiosensitivity of endothelial cells in subsequent irradiations. This indicates that miRNAs are part of the innate response mechanism of the endothelium to radiation.

## Background

MicroRNAs (miRNAs, miRs) are a group of short, non-coding RNAs (~22 nucleotides in length) that have emerged as important (negative) regulators of gene expression. It has been shown that up to 100-200 mRNAs can be repressed by one miRNA [1]. These molecules are considered key players in a variety of processes ranging from development, proliferation, morphogenesis and differentiation to cancer and apoptosis [2,3].

Roles of microRNAs in cancer development have been documented in several studies [4,5]. Typically, miRNAs involved in tumorigenesis are deregulated, and this deregulation is believed to alter the expression of protein-coding mRNA, thereby favoring uncontrolled tumor cell growth. The deregulation can be an under- or overexpression, suggesting that miRNAs may function as tumor suppressors or as oncogenes. The involvement of miRNAs in tumorigenesis is not the only topic of investigation. In addition the expression patterns of these regulators by cancer treatment modalities such as radiotherapy or chemotherapy are increasingly recognized. It has been shown for cancer cells that the expression of miRNAs may vary depending on parameters like cell type, post-radiation time and radiation dose [6-8].

The tumor vessel system, and in turn endothelial cells as the characteristic parts of the vessel system, constitute critical targets for radiotherapy of tumors. However, to our best knowledge, the regulation of miRNAs in endothelial cells (EC) after radiation has not been investigated to date. EC are sensitive to ionizing radiation in proliferation and clonogenic assays in vitro and in vivo [9] and may constitute critical targets in normal tissue such as in the gut microvasculature [10]. In contrast, EC are also stimulated by radiation-induced indirect pro-angiogenic factor production including VEGF and bFGF [9,11]. Further ionizing radiation potently causes DNA damage, which has been shown to induce miRNA expression via the p53 network [12]. Here we investigated the miRNA response in EC after ionizing radiation. To this end, human EC were irradiated and radiation-induced alterations of miRNA levels were analyzed by miRNA microarrays. The most stringently regulated miRNAs were then further analyzed. The effects of miRNA overexpression or inhibition were determined in functional assays including clonogenic assays with and without radiation in order to examine if the altered miRNA levels affected EC response to radiation.

## Methods

### Cell culture

Human dermal microvascular endothelial cells (HDMEC; PromoCell, Heidelberg, Germany) were cultured in modified PromoCell medium (for ref. see [13]) for optimal growth results. Cells were cultured up to passage 7; for transfection cells of passage 3 to 5 were used.

### Isolation of RNA

Cells were seeded in culture flasks until confluency of ~70% before 2 Gy photon irradiation (RT, 6 MeV; LINAC, Siemens). After RT they were transferred back to the incubator and after 6 hours lysed and stored at -80°C. Non-irradiated cells were used as controls. RNA was isolated from HDMEC using TRIzol LS reagent (Invitrogen, Karlsruhe, Germany) (3 biological replicates each for RT and control) as described by the manufacturers. Quality and quantity of isolated RNA were checked using Lab on Chip technology on Agilent 2100 bioanalyzer (Agilent technologies, CA, USA) and a Nanodrop spectrophotometer (ND-1000; Nanodrop technologies, DE, USA).

### Locked nucleic acid (LNA) -based miRNA microarrays and data analysis

RNA samples from the three biological replicates were used for LNA-based array analysis. miRNA expression profiling was performed using a microarray platform which is based on locked nucleic acid (LNA)-modified capture probes which are immobilized on the chip surface. For detailed protocol and further details see Castoldi et al. [14] and Exiqon (http://www.exiqon.com). 361 miRNAs, including 315 human miRNAs were spotted in quadruplicates on the slides (see Additional file 1). Slides were scanned using the Genepix 4000B scanner (Axon instruments). Data analyses were done using 'Microsoft Excel' software and the 'SUMO' software package for microarray data evaluation (http://www.oncoexpress.de/software/sumo/). For data normalization we developed a step-wise approach: First, normalization was performed on the background-subtracted mean intensity values against the intensity of the U6 snRNA spots on each chip. After this thresholding, the data underwent a two-class t-test. We then created a short-list of differentially expressed miRNAs as described in 'Results'. Microarray data were deposited in 'ArrayExpress' (accession no.: E-TABM-617).

### Transfection

Transfection of primary HDMEC was performed using the siPORT Amine (Ambion, Texas, USA) transfection reagent. Transfection efficiency was analyzed using a GAPDH assay (KDalert, Ambion). The efficiency of transfection conditions was 30-50%. Furthermore a miR-1 transfection test system (Ambion) was used, which is known to down-regulate the PTK9 mRNA in human cells. Expression of PTK9 was measured by real-time PCR to verify our transfection conditions (see Additional file 2). In the experiments miRNA precursor (pre-miR) or inhibitor (anti-miR) molecules or the appropriate negative control molecules were added to the cells in a final concentration of 50 nM. The following pre- and anti-miRs were used: hsa-let-7g, hsa-miR-125a, hsa-miR-127, hsa-miR-148b, hsa-miR-189, hsa-miR-20a, pre-miR negative control #1, and anti-miR negative control #1 (all purchased from Ambion).

### Clonogenic survival assay

HDMEC were pre-plated in 25 cm^2 ^cell culture flasks; cell numbers varied depending on the treatment. In experimental settings with transfection medium and/or RT cell numbers were raised. After one day the transfection mixture was added for 6 hours, then cells were re-fed with normal growth medium. After 24 hours cells were irradiated with 2 Gy (6 MeV X-rays; LINAC, Siemens) and then returned to the incubator for 8-10 days. Untreated cells served as growth controls. For evaluation the number of counted colonies was normalized to the amount of pre-plated cells. At the end of the incubation period cells were stained with crystal violet (Sigma-Aldrich, Germany) and colonies were counted. All conditions were done in triplicate, the survival experiments for each miRNA were repeated three times.

### Proliferation assay

The proliferation rate of cells was determined using a calcein assay (PromoKine, Heidelberg, Germany). The assay was performed in a 96 well-plate format. 2500 endothelial cells were seeded per well, after 1 day they were transfected for 6 hours, cultured with normal growth medium and incubated for another 24 hours. Then the cells were irradiated with 2 or 10 Gy - while controls were non-irradiated - and incubated for 3 days. Intracellular fluorescent calcein is directly proportional to the number of living cells and was measured using a plate reader (CytoFluor, PerSeptive Biosystems) with 485 nm excitation and 530 nm emission filters.

### Statistical analysis

Statistical data evaluation was performed using two-tailed t-tests or in case of multiple comparisons using ANOVA along with Fisher's least significance difference test. The significance level was P < 0.05.

## Results

### miRNA array data

**Figure 1:**
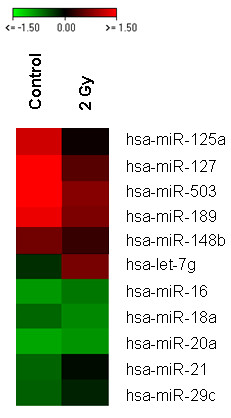
**Differentially expressed miRNAs in HDMEC**. The figure includes those miRNAs which are listed in Tab. 1. The heat-map was generated via a t-test of microarray data of radiation treated versus untreated cells. Each column represents the medium value of three biological replicates. Colours represent log2 values from -1.5 to 1.5.

The miRNA expression profile of HDMEC six hours after 2 Gy radiation treatment (RT) was analyzed using oligo-microarrays. For data analysis we generated a short-list of the most stringently regulated miRNAs (p-value < 0.05) using a t-test and identified 83 genes. Because each miRNA was spotted four times on a chip, we selected those which were present three or four times in our short-list and which had a minimum spot intensity value of 1000. Finally, we identified 11 miRNAs from the t-test and considered them as regulated by irradiation at the dose of 2 Gy (Tab. 1). In terms of 'fold change' the regulation revealed small but statistically significant values (p-value of 0.05 or lower) between 0.5- and 1.5-fold (Fig. 1).

**Table 1:** miRNA species altered in HDMEC in response to radiation (2 Gy)

	*fold change*	*p-value (2-class t-test)*
**let-7g**	1.56	0.020
**miR-16**	1.35	0.028
**miR-18a**	0.51	0.036
**miR-125a**	0.53	0.008
**miR-127**	0.56	0.063
**miR-148b**	0.53	0.040
**miR-189**	0.47	0.049
**miR-20a**	1.51	0.025
**miR-21**	1.49	0.038
**miR-29c**	1.66	0.005
**miR-503**	0.49	0.030

Microarray data of radiation treated versus untreated cells; t-test was performed with 3 biological replicates each. Each 'fold change' value represents a medium of 3-4 individual spot values.

### Endothelial cell response to miRNA overexpression and inhibition

#### Clonogenic survival assays

Out of the microRNA list from the microarrays we selected six miRNAs (let-7g, miR-125a, miR-127, miR-148b, miR-189, and miR-20a) for further functional analysis.

**Figure 2:**
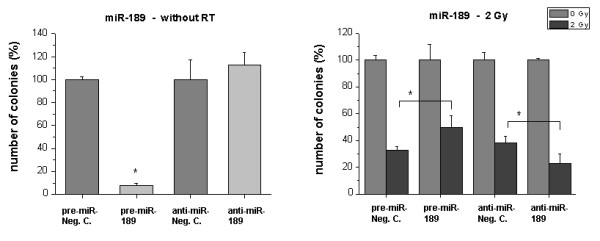
**Clonogenic survival of HDMEC after transfection with miR-189 precursor or inhibitor**. Cells were treated as described in 'Methods'. *Bar charts: *Left sided: Influence of the transfected molecules on clonogenic survival without RT. Negative controls were set 100%. Right sided: Survival of irradiated cells (2 Gy) after transfection with the miRNA precursor (pre-miR) or inhibitor (anti-miR). Only miR transfected samples without irradiation were set 100%. Bars: Mean (n = 3) with SD. *: P < 0.05 versus the pre-miR or anti-miR negative control.

We found that the overexpression or inhibition, respectively, of miR-189, let-7g and miR-20a showed the strongest effects on functional cell behavior. In comparison to respective unspecific control molecules, miR-189 precursor strongly inhibited clonogenic survival in HDMEC (P < 0.05) (Fig. 2). In contrast, we observed different effects after additional radiation of the cells: Pretreatment of cells with miR-189 precursor prior to a 2 Gy radiation treatment caused an increase of clonogenic survival, while a pretreatment with miR-189 inhibitor caused a reduction in clonogenic survival (P < 0.05).

**Figure 3:**
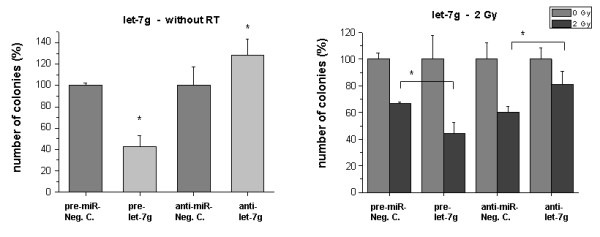
**Clonogenic survival of HDMEC after transfection with let-7g precursor or inhibitor**. Cells were treated as described in 'Methods'. *Bar charts: *Left sided: Influence of the transfected molecules on clonogenic survival without RT. Negative controls were set 100%. Right sided: Survival of irradiated cells (2 Gy) after transfection with the miRNA precursor (pre-miR) or inhibitor (anti-miR). Only miR transfected samples without irradiation were set 100%. Bars: Mean (n = 3) with SD. *: P < 0.05 versus the pre-miR or anti-miR negative control.

We also found that overexpression and inhibition, respectively, of let-7g had similar effects on clonogenic survival like miR-189 (Fig. 3). The addition of pre-let-7g caused a dramatic reduction by ~50% of clones. Anti-let-7g had the opposite effect and enhanced the clonogenic survival (P < 0.05). After irradiation the same pattern was observed: Overexpression of let-7g further reduced clonogenic survival of cells irradiated with 2 Gy vs. irradiated controls, while let-7g inhibition significantly improved clonogenic survival (P < 0.05) vs. irradiated controls (Fig. 3, right panel).

**Figure 4:**
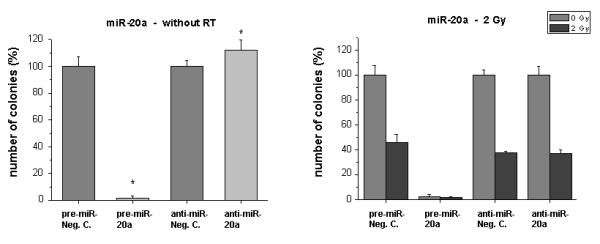
**Clonogenic survival of HDMEC after transfection with miR-20a precursor or inhibitor**. Cells were treated as described in 'Methods'. *Bar charts: *Left sided: Influence of the transfected molecules on clonogenic survival without RT. Negative controls were set 100%. Right sided: Survival of irradiated cells (2 Gy) after transfection with the miRNA precursor (pre-miR) or inhibitor (anti-miR). Only miR transfected samples without irradiation were set 100%, except for pre-miR-20a. Bars: Mean (n = 3) with SD. *: P < 0.05 versus the pre-miR or anti-miR negative control.

Furthermore, we measured in non-irradiated EC a strong inhibition of clonogenic survival by pre-miR-20a, while the downregulation of the miR-20a level increased the number of clones (P < 0.05) (Fig. 4). Although the direct functional effects of miR-20a over- or underexpression were strong, the clonogenicity upon irradiation was not markedly affected.

**Figure 5:**
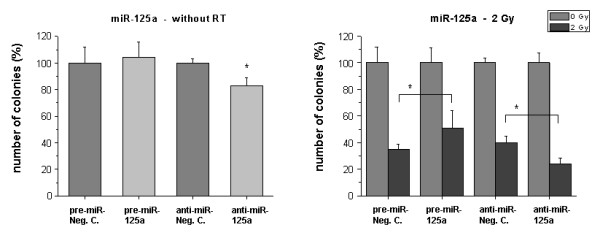
**Clonogenic survival of HDMEC after transfection with miR-125a precursor or inhibitor**. Cells were treated as described in 'Methods'. *Bar charts: *Left sided: Influence of the transfected molecules on clonogenic survival without RT. Negative controls were set 100%. Right sided: Survival of irradiated cells (2 Gy) after transfection with the miRNA precursor (pre-miR) or inhibitor (anti-miR). Only miR transfected samples without irradiation were set 100%. Bars: Mean (n = 3) with SD. *: P < 0.05 versus the pre-miR or anti-miR negative control.

**Figure 6:**
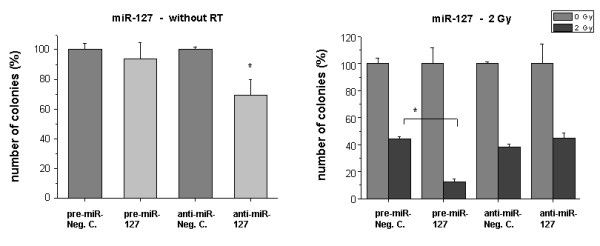
**Clonogenic survival of HDMEC after transfection with miR-127 precursor or inhibitor**. Cells were treated as described in 'Methods'. *Bar charts: *Left sided: Influence of the transfected molecules on clonogenic survival without RT. Negative controls were set 100%. Right sided: Survival of irradiated cells (2 Gy) after transfection with the miRNA precursor (pre-miR) or inhibitor (anti-miR). Only miR transfected samples without irradiation were set 100%. Bars: Mean (n = 3) with SD. *: P < 0.05 versus the pre-miR or anti-miR negative control.

For miR-125a and -127 we found that the downregulation of the miRNA levels caused a significant reduction (P < 0.05) of clonogenic survival (Figs. 5 and 6, left panels). The overexpression of miR-125a or miR-127 showed no marked effects on clonogenicity per se. However, the altered expression significantly influenced the response to radiation: Pre-miR-125a enhanced the number of clones compared to irradiated mock control cells, while anti-miR-125a reduced clonogenic survival (P < 0.05) (Fig. 5, right panel). miR-127 overexpression had a strong negative effect on clonogenic survival in 2 Gy treated cells (P < 0.05) (Fig. 6, right panel).

**Figure 7:**
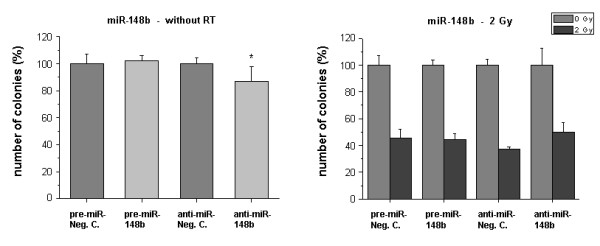
**Clonogenic survival of HDMEC after transfection with miR-148b precursor or inhibitor**. Cells were treated as described in 'Methods'. *Bar charts: *Left sided: Influence of the transfected molecules on clonogenic survival without RT. Negative controls were set 100%. Right sided: Survival of irradiated cells (2 Gy) after transfection with the miRNA precursor (pre-miR) or inhibitor (anti-miR). Only miR transfected samples without irradiation were set 100%. Bars: Mean (n = 3) with SD. *: P < 0.05 versus the pre-miR or anti-miR negative control.

In the experiments with miR-148b we mainly observed an inhibitory effect of anti-miR-148b on clonogenic survival in non-irradiated cells (Fig. 7).

#### Cell proliferation/viability assays

**Figure 8:**
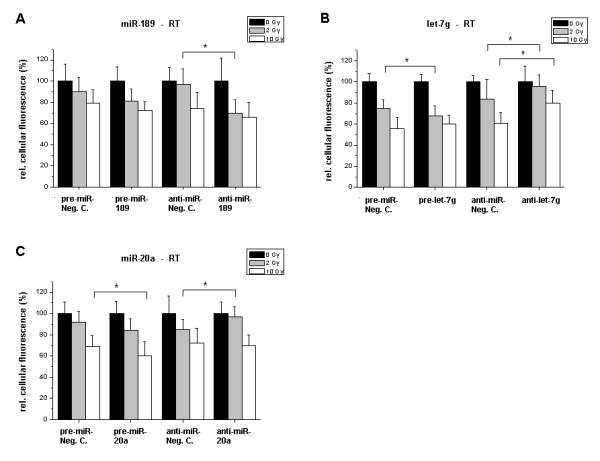
**Proliferation assay of irradiated cells**. HDMEC were treated as described in 'Methods'. Panels A-C show the proliferation data of cells pre-treated with precursor or inhibitor molecules of miR-189, let-7g and miR-20a. The bar charts present the mean proliferation of HDMEC after irradiation, dependent on the pre-treatment. Fluorescence values are set in percentage related to non-irradiated cells (100%). *Bars: *Mean (n = 24) with SD. *: P < 0.05 versus the pre-miR or anti-miR negative control.

Aside from clonogenic survival we also studied cell proliferation as a functional endpoint. In Fig. 8 the effects of ionizing radiation after transfection with precursors or inhibitors of miR-189, let-7g and miR-20a are shown. As for clonogenicity, overexpression or inhibition of miRNAs significantly altered endothelial cell properties in response to radiation. The viability of the cells after RT was checked by light microscopy. While EC treated with 2 Gy apparently did not show visible alterations compared to the untreated cells, cells treated with 10 Gy showed typical signs of cell stress, like cytoplasmic contraction, but were still adherent. After 10 Gy radiation treatment the number of cells always was somewhat reduced compared to the 2 Gy treatment. The degree of reduction was dependent on the various transfection treatments.

While the overexpression of miR-189 had no significant effect on the proliferation of irradiated cells compared to the negative control (Fig. 8A) proliferation decreased further after transfection with anti-miR-189 vs. the respective negative control (P < 0.05). This alteration was in particular found at 2 Gy. At the high radiation dose of 10 Gy this differential effect was hardly present any longer.

As shown in Fig. 8B, overexpression of let-7g further reduced cell number after irradiation with 2 Gy in comparison to the control miRNA. In contrast, the inhibition of let-7g attenuated the growth inhibitory effect of the radiation treatment at the dose of 2 Gy. This prosurvival effect in endothelial cells of anti-let-7g was also present in the 10 Gy radiation setting (P < 0.05).

In the case of miR-20a the effects on clonogenicity and proliferation were remarkably different: Compared to the strong inhibitory effect of miR-20a precursor on clonogenic survival the proliferation inhibition of irradiated cells was much more moderate, but significant (Fig. 8C). miR-20a inhibition showed the opposite effect in cells with 2 Gy RT.

Taken together, as shown in both proliferation and clonogenicity assays, the overexpression or inhibition of radiation-inducible miRNAs markedly changes functional cell behavior, and alters the intrinsic properties of endothelial cells. Moreover, in both assays the functional radiosensitivity of endothelial cells is modified after irradiation by altered radiation-induced miRNAs.

## Discussion

The expression profiling by microarray showed that irradiation at the clinically relevant dose of 2 Gy induced significant changes in miRNA levels in human dermal microvascular endothelial cells (HDMEC). Six microRNAs (miRs) were chosen for subsequent functional analyses (let-7g, miR-125a, miR-127, miR-148b, miR-189, and miR-20a).

The proliferation and clonogenic assays documented that overexpression or inhibition of the here identified miRNAs is capable of reducing or enhancing endothelial cell proliferation and/or clonogenic survival. Moreover, we also found that overexpression or inhibition of selected miRNAs either enhanced or attenuated the radiation-induced reduction of clonogenicity or proliferation of HDMEC. This indicates that changes in radiation-induced miRNA expression alter the intrinsic functional cell properties and suggest that the radiosensitivity of endothelial cells itself is modified after irradiation. We conclude that radiation-induced miRNA expression alterations may play important roles in the desired and, potentially, also in side effects of radiotherapy of cancer and other applications of ionizing radiation.

One of the up-regulated miRNAs in response to radiation was a member of the let-7 family (let-7g). Other let-7 family members were not regulated upon radiation treatment (RT) or slightly up-regulated like let-7d and let-7f (see Additional file 3). A role of let-7 in cell growth has been described in normal and lung cancer cells, in which let-7 is down-regulated [15]. Let-7 negatively regulates human Ras genes and it was reported that it is a negative regulator of cell proliferation pathways in human cells [16]. An alteration in the expression of let-7 miRNAs in response to radiation was recently shown in human fibroblasts [17]. Furthermore, a role of several let-7 miRNA family members for radiation sensitivity in lung cancer cells was reported by Weidhaas et al. The authors showed that overexpression of let-7g protected A549 cells from radiation. Corresponding to the let-7g up-regulation in our irradiated endothelial cells we found a reduction of clonogenic survival by overexpression of the miRNA. We found enhanced survival by inhibition of let-7g both in untreated and in irradiated cells. The data suggest that let-7g negatively regulates EC growth and furthermore sensitizes them to radiation. Since radiation up-regulates let-7g, the data also indicate that the miRNA up-regulation is correlatively or causatively associated with the direct anti-endothelial radiation effect as determined by clonogenic survival and proliferation inhibition.

miR-20a was also found to be up-regulated after radiation treatment. This microRNA sequence lies within the cluster miR-17-92, which is up-regulated in several human tumor types including lung, pancreas, prostate and colon cancer [18]. Matsubara et al. could show that the inhibition of miR-20a can induce apoptosis in lung cancer cells over-expressing the miR-17-92 cluster [19]. Furthermore, miR-20a is involved in cell cycle progression [20]. Our own cell-based assays clearly show that miR-20a overexpression dramatically inhibits clonogenic survival, while the inhibition of the miRNA increased survival rates. Again, since radiation was clearly found to up-regulate miR-20a, this microRNA is another potential candidate in our system linking functional cell death with effects of radiation. Interestingly, our data showed that the radiation sensitivity itself in endothelial cells does not appear to be markedly dependent on the expression level of miR-20a.

The microRNAs miR-189, -125a, -127 and 148b were all found to be down-regulated after RT of 2 Gy. In the case of miR-189 the functional experiments revealed interesting opposite effects on cell growth with and without radiation: In survival assays we observed a strong decrease of clonogenic survival after overexpression of miR-189. In contrast, versus additional radiation as control sample, miR-189 over-expression increased clone number and miR-189 inhibition decreased clone number. These data suggest that miR-189 expression per se has negative effects on clonogenic survival and proliferation of endothelial cells. Radiation decreases the expression levels, which suggests that the anti-endothelial effects are associated with down-regulated miRNA expression. Moreover, and in line with these functional findings, miR-189 up-regulation seems to exert protective effects against radiation with an attenuation of radiation-induced growth inhibition.

In functional assays with miR-125a, -127 and -148b we observed weaker effects of miR overexpression or inhibition. According to our own results miR-125a is also differentially expressed in human fibroblasts by hydrogen peroxide (H_2_O_2_), which is like ionizing radiation a stressor for cells [17]. When changing levels of miR-125a we found a decrease of clonogenic survival upon inhibition. Similar to miR-189, miR-125a had a positive effect on endothelial clonogenic survival after irradiation. Accordingly, we found that inhibition of miR-125a had the respective negative effects, comparable to non-RT conditions.

miR-127 also was found to be down-regulated in irradiated cells. Originally it had been described as a putative tumor suppressor. It is silenced in tumor cells, which causes the overexpression of the proto-oncogene bcl-6 [21]. Likewise, we also found that the inhibition of miRNA-127 reduced clonogenic survival (and proliferation; data not shown), suggesting that the anti-endothelial radiation effects are associated with down-regulated miR-127 levels. Conversely, over-expressed miR-127 enhanced radiation sensitivity in clonogenic assays. Perhaps the executed signaling pathways are dependent on the expression levels of other parameters suggesting a functional 'switch' role of miRNA-127. Another explanation would be that the downregulation of miRNA-127 after radiation is not functionally in line but rather part of a negative feedback mechanism. Moreover, a dual role of miRNAs has recently been described, showing that a miRNA can repress or enhance mRNA translation, depending on the state of the cell cycle [22].

Further, it has been described that ionizing radiation also may have dual roles with respect to endothelial cells, angiogenesis and the microenvironment: while radiation has dominantly direct anti-endothelial effects, it may also convey indirect pro-angiogenic effects with up-regulation of VEGF, PDGF or AKT signaling in endothelium. One might speculate that miR-127 is involved in such or similar pro-survival mechanisms [13,23].

In the case of miR-148b irradiation down-regulated expression levels. miR-148b inhibition itself slightly reduced clonogenic growth. In contrast, and similarly to the findings for miRNA-127, miR-148b inhibition might favor survival under radiation conditions.

## Conclusion

Taken together we have shown here that ionizing radiation of HDMECs induces alterations of miRNA levels with up- as well as down-regulations. We found that especially miR-189, let-7g, and miR-20a seem to play a role in endothelial cell clonogenic survival and/or proliferation, and to a weaker extend also miR-125a, -127, and -148b. Furthermore, we show that alterations of miRNA levels modify EC radiosensitivity. While in particular miR-189 and miR-125a have a protective effect on endothelial cells, miR-127 and let-7g enhance their sensitivity to irradiation. Since we performed our studies in human primary endothelium as effector cells of angiogenesis and tumor angiogenesis [24], it is conceivable that the miRNAs identified here may also influence angiogenesis in vivo in normal tissue and tumors. Moreover, with respect to carcinogenesis and cancer therapy, radiation effects might be conveyed, modified or associated with differential regulations of miRNAs. Therefore, the modulation of miRNA levels may have implications for anticancer treatments, in particular for radiotherapy alone and in combination with drugs [25].

## Competing interests

The authors declare that they have no competing interests.

## Authors' contributions

MW-E designed experiments, performed experiments, analyzed data and wrote the manuscript. CS generated software for data evaluation and bio-statistical analyses. AA designed experiments and wrote the manuscript. UW designed experiments and analyzed data. PH designed experiments, analyzed data and wrote the manuscript. All authors read and approved the final manuscript.

## Supplementary Material

Additional file 1 **List of miRNAs on the microarrays**. The file contains a list of miRNAs (Reporter ID, miRBase Entry and sequence) which were spotted on the microarrays. Click here for file

Additional file 2 **Testing of various transfection conditions for functional assays**. HDMEC were transfected with miR-1 (Ambion), then RNA was isolated and the expression of the PTK9 gene was measured by real-time PCR (Roche Light Cycler 480). miR-1 is known to down-regulate the PTK9 mRNA in human cells. Bar chart: Expression ratio of the PTK9 gene versus a reference gene (18S rRNA). Click here for file

Additional file 3 **Microarray data of let-7 family members from HDMEC**. Microarrays were performed of radiation treated (2 Gy) versus untreated cells; t-test was done with 3 biological replicates each. Each 'fold change' value represents the mean of all spot values. Click here for file
