# Supplementary material for: MicroRNA expression after ionizing radiation in human endothelial cells
Source: Radiat Oncol. 2010 Mar 26;5:25. doi: 10.1186/1748-717X-5-25 (PMC2859352; doi:10.1186/1748-717X-5-25)
Supplement: Additional file 2 — Testing of various transfection conditions for functional assays. HDMEC were transfected with miR-1 (Ambion), then RNA was isolated and the expression of the PTK9 gene was measured by real-time PCR (Roche Light Cycler 480). miR-1 is known to down-regulate the PTK9 mRNA in human cells. Bar chart: Expression ratio of the PTK9 gene versus a reference gene (18S rRNA). [file 1748-717X-5-25-S2.PPT]

## Slide 1
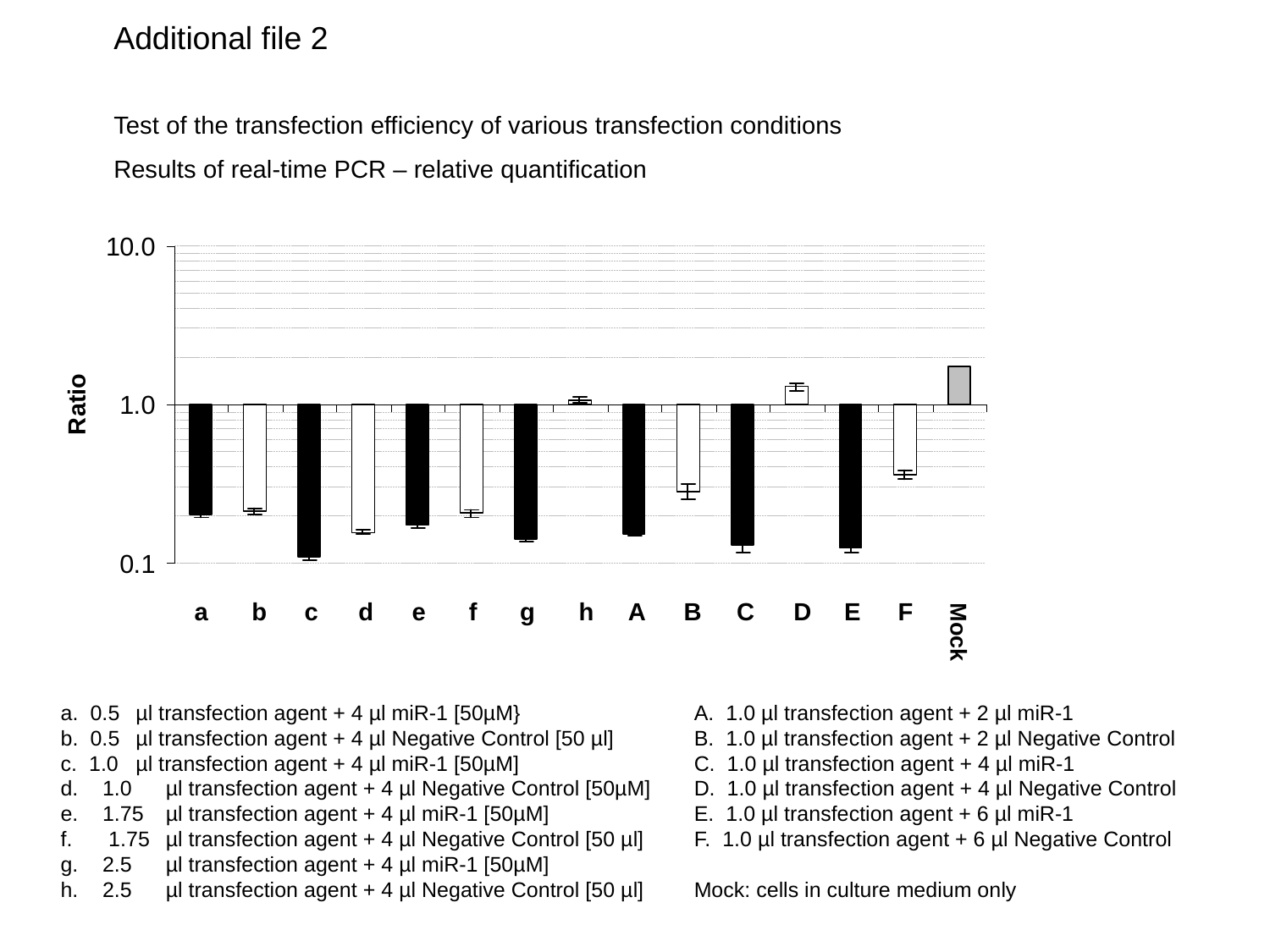

Additional file 2
Test of the transfection efficiency of various transfection conditions
Results of real-time PCR – relative quantification
a
b
c
d
e
f
g
h
A
B
C
D
E
F
Mock
a. 0.5	µl transfection agent + 4 µl miR-1 [50µM}
b. 0.5	µl transfection agent + 4 µl Negative Control [50 µl]
c. 1.0	µl transfection agent + 4 µl miR-1 [50µM]
 1.0	µl transfection agent + 4 µl Negative Control [50µM]
 1.75	µl transfection agent + 4 µl miR-1 [50µM]
 1.75	µl transfection agent + 4 µl Negative Control [50 µl]
 2.5	µl transfection agent + 4 µl miR-1 [50µM]
 2.5	µl transfection agent + 4 µl Negative Control [50 µl]
A. 1.0 µl transfection agent + 2 µl miR-1
B. 1.0 µl transfection agent + 2 µl Negative Control
C. 1.0 µl transfection agent + 4 µl miR-1
D. 1.0 µl transfection agent + 4 µl Negative Control
E. 1.0 µl transfection agent + 6 µl miR-1
F. 1.0 µl transfection agent + 6 µl Negative Control
Mock: cells in culture medium only
