# Supplementary material for: MicroRNA expression after ionizing radiation in human endothelial cells
Source: Radiat Oncol. 2010 Mar 26;5:25. doi: 10.1186/1748-717X-5-25 (PMC2859352; doi:10.1186/1748-717X-5-25)
Supplement: Additional file 3 — Microarray data of let-7 family members from HDMEC. Microarrays were performed of radiation treated (2 Gy) versus untreated cells; t-test was done with 3 biological replicates each. Each 'fold change' value represents the mean of all spot values. [file 1748-717X-5-25-S3.DOC]

**Alteration of let-7 family members in response to radiation (2 Gy)**

*fold change p-value (2-class t-test)*

**let-7a** 0.99 0.22

**let-7b** 0.96 0.39

**let-7c** 1.04 0.27

**let-7d** 1.23 0.05

**let-7e** 0.96 0.31

**let-7f** 1.34 0.07

**let-7g** 1.56 0.02
